# Systemic Infection by Non-*albicans Candida* Species Affects the Development of a Murine Model of Multiple Sclerosis

**DOI:** 10.3390/jof8040386

**Published:** 2022-04-10

**Authors:** Thais Fernanda de Campos Fraga-Silva, Natália Munhoz-Alves, Luiza Ayumi Nishiyama Mimura, Larissa Ragozo Cardoso de Oliveira, Lívia Mara Alves Figueiredo-Godoi, Maíra Terra Garcia, Evelyn Silva Oliveira, Larissa Lumi Watanabe Ishikawa, Sofia Fernanda Gonçalves Zorzella-Pezavento, Vânia Luiza Deperon Bonato, Juliana Campos Junqueira, Eduardo Bagagli, Alexandrina Sartori

**Affiliations:** 1 Department of Chemistry and Biological Sciences, Institute of Biosciences, São Paulo State University (UNESP), Botucatu 18618-689, Brazil; natalia.mnhz@gmail.com (N.M.-A.); luizamimura@gmail.com (L.A.N.M.); soliveira.evelyn@gmail.com (E.S.O.); larissalumi@gmail.com (L.L.W.I.); szorzella@yahoo.com.br (S.F.G.Z.-P.); eduardo.bagagli@unesp.br (E.B.); alexandrina.sartori@unesp.br (A.S.); 2 Postgraduate Program in Tropical Disease, Botucatu Medical School, São Paulo State University (UNESP), Botucatu 18618-687, Brazil; larissa.ragozo@unesp.br; 3 Institute of Science and Technology, São Paulo State University (UNESP), Sao Jose dos Campos 12245-000, Brazil; livia_mafigueiredo@yahoo.com.br (L.M.A.F.-G.); maira.garcia@unesp.br (M.T.G.); juliana.junqueira@unesp.br (J.C.J.); 4 Department of Biochemistry and Immunology, Ribeirao Preto Medical School, University of Sao Paulo (USP), Ribeirao Preto 14049-900, Brazil; vlbonato@fmrp.usp.br

**Keywords:** non-*albicans Candida* spp., *Galleria mellonella*, central nervous system, experimental autoimmune encephalomyelitis, microglia

## Abstract

Candidiasis may affect the central nervous system (CNS), and although *Candida albicans* is predominant, non-*albicans Candida* species can also be associated with CNS infections. Some studies have suggested that *Candida* infections could increase the odds of multiple sclerosis (MS) development. In this context, we investigated whether systemic infection by non-*albicans Candida* species would affect, clinically or immunologically, the severity of experimental autoimmune encephalomyelitis (EAE), which is an animal model used to study MS. For this, a strain of *C. glabrata*, *C. krusei*, and *C. parapsilosis* was selected and characterized using different in vitro and in vivo models. In these analysis, all the strains exhibited the ability to form biofilms, produce proteolytic enzymes, and cause systemic infections in *Galleria mellonella*, with *C. glabrata* being the most virulent species. Next, C57BL/6 mice were infected with strains of *C. glabrata*, *C. krusei*, or *C. parapsilosis*, and 3 days later were immunized with myelin oligodendrocyte glycoprotein to develop EAE. Mice from EAE groups previously infected with *C. glabrata* and *C. krusei* developed more severe and more prevalent paralysis, while mice from the EAE group infected with *C. parapsilosis* developed a disease comparable to non-infected EAE mice. Disease aggravation by *C. glabrata* and *C. krusei* strains was concomitant to increased IL-17 and IFN-γ production by splenic cells stimulated with fungi-derived antigens and with increased percentage of T lymphocytes and myeloid cells in the CNS. Analysis of interaction with BV-2 microglial cell line also revealed differences among these strains, in which *C. krusei* was the strongest activator of microglia concerning the expression of MHC II and CD40 and pro-inflammatory cytokine production. Altogether, these results indicated that the three non-*albicans Candida* strains were similarly able to reach the CNS but distinct in terms of their effect over EAE development. Whereas *C. glabrata* and *C. Krusei* aggravated the development of EAE, *C. parapsilosis* did not affect its severity. Disease worsening was partially associated to virulence factors in *C. glabrata* and to a strong activation of microglia in *C. krusei* infection. In conclusion, systemic infections by non-*albicans Candida* strains exerted influence on the experimental autoimmune encephalomyelitis in both immunological and clinical aspects, emphasizing their possible relevance in MS development.

## 1. Introduction

The genus *Candida* comprises pleomorphic fungi commonly found as constituents of the microbiota in the human gastrointestinal and genitourinary tracts and opportunistic pathogens under predisposing conditions, such as immunosuppression [1,2]. While acting as pathogens, *Candida* spp. can cause cutaneous, mucosal, and systemic infections [2,3]. The latter is usually related to forms of invasive candidiasis, which are mainly represented by infections in the blood and may compromise vital organs and systems such as the lungs, kidneys, bones, heart, and central nervous system (CNS) [4]. In this regard, CNS fungal infections include *Candida* infections [5]; whilst *C. albicans* is predominant, non-*albicans Candida* species are also able to reach the CNS [6]. Accumulating data indicate the predominance of five species in *Candida* infections: *C. albicans*, *C. glabrata*, *C. parapsilosis*, *C. krusei*, and *C. tropicalis* [7,8,9,10]. Although *C. albicans* remains the most frequent species, the incidence of non-*albicans Candida* infections has been increasing in the last years [11,12,13].

In addition to the infectious disease itself, these fungi have also been associated with autoimmunity induction or aggravation [14]. The potential mechanism of this involvement has been partially deciphered and can include the susceptibility to fungal infections, their dissemination through the circulatory system, the activation of antigen-presenting cells, the subsequent imbalance between T helper 17 (Th17) and T regulatory lymphocytes, and the induction of inflammation and tissue damage [15]. The possible contribution of fungal infections to multiple sclerosis (MS) development has been more lately proposed and investigated. MS is a chronic inflammatory disease characterized by an autoimmune response-mediated primarily by T lymphocytes, against components of the CNS, mainly myelin sheath self-antigens, which causes demyelination, axonal damage, and loss of neuronal function [16,17,18]. The deleterious effects of fungi infections in the context of MS have been attributed to some mechanisms such as, for example, the cross-reactivity of the fungus with human tissues, including the brain [19], the production of toxins that could release autoantigens through toxic effects on astrocytes and oligodendrocytes [20], and the direct pro-inflammatory effect of these fungi in the CNS, with subsequent activation of microglial cells [21]. In this regard, we recently demonstrated that the systemic inoculation of gliotoxin in mice aggravated the encephalomyelitis by increasing the neuroinflammatory process [22].

MS is believed to be triggered by a complex interaction among several genetic and environmental factors, including various infectious agents such as fungi, as mentioned above. The hypothesis concerning the contribution of *Candida* spp. infections in MS pathogenesis has been sustained by some clinical and experimental evidences. Studies demonstrated, for example, the presence of high levels of anti-*Candida* antibodies and *Candida* antigens (proteins) in the blood and cerebrospinal fluid of MS patients [23,24,25,26]. In addition, the oral colonization of *Candida* spp. in MS patients was higher than in healthy individuals [27], and *C. albicans* isolated from MS patients presented a higher specific enzyme activity compared to *C. albicans* isolated from healthy individuals and had a positive correlation with the severity of MS [28]. Using the experimental autoimmune encephalomyelitis (EAE) model, which is extensively employed to study the immunopathogenesis of MS [29,30,31], our research group previously demonstrated that *C. albicans* spreads to the CNS and aggravates the development of EAE due to the increased production of encephalitogenic cytokines [32]. We also described that, similar to *C. albicans* [33], *C. glabrata*, *C. krusei*, and *C. parapsilosis* were all able to disseminate to the CNS and promote local inflammation in both immunocompetent and immunosuppressed mice [34].

In this context, we hypothesized that non-*albicans Candida* species could also interfere with the development of MS. To test this possibility, we evaluated the effect of infections with strains of *C. glabrata*, *C. krusei*, and *C. parapsilosis* on EAE evolution. To determine the possible mechanisms involved in disease severity, we employed different in vivo and in vitro models of *Candida* infections.

## 2. Materials and Methods

### 2.1. Non-albicans Candida Strains

Two strains from the ATCC collection, *C. krusei* (ATCC 6258, GenBank KC601854.1) and *C. parapsilosis* (ATCC 90018, GenBank KU729146.1), and one strain, *C. glabrata* (H-3479, GenBank KX774384.1), originally isolated from a blood culture of a patient admitted to the University Hospital of the Botucatu Medical School (São Paulo State University, Botucatu, SP, Brazil), were used. The three strains, which were being kept frozen in 10% glycerol–yeast extract–peptone medium, were grown on Sabouraud dextrose agar (Becton Dickinson and Company, Sparks, MD, USA) plates for 24 h at 37 °C, and then carefully washed and resuspended in PBS before experimental infections.

### 2.2. Animal Models

Larvae of *Galleria mellonella* (Linnaeus, 1758) were reared and maintained at the Laboratory of Microbiology and Immunology of the Institute of Science and Technology (São Paulo State University, São José dos Campos, SP, Brazil) until final larval stage (250–300 mg), as previously described for use in a *Candida albicans* model of infection [35]. Adult female (6 weeks old) *Mus musculus* Linnaeus, 1758, were purchased from the animal facility of the University of São Paulo (Ribeirão Preto, SP, Brazil) and kept in a specific pathogen-free environment in mini-isolators (Alesco, Monte Mor, SP, Brazil) containing a maximum of five mice per cage. Animals were maintained with free and sterile feed and water in the Animal House of the Department of Chemical and Biological Sciences of the Institute of Biosciences (São Paulo State University, Botucatu, Brazil). All procedures with mice were performed in conformity with the local Ethics Committee on Use of Animals (São Paulo State University, Botucatu, SP, Brazil, protocol number 853).

### 2.3. Experimental Design

**Figure 1:**
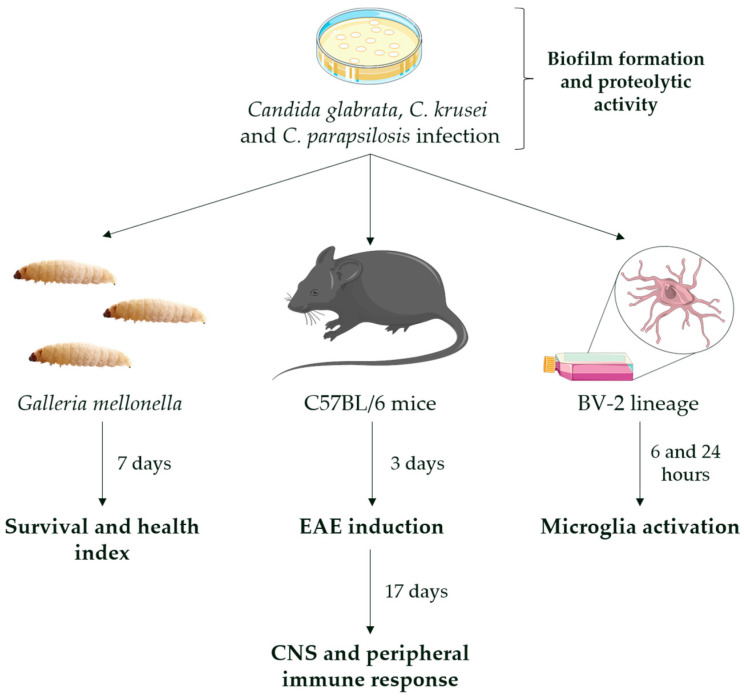
Experimental design. Yeasts of *C. glabrata*, *C. krusei*, and *C. parapsilosis* were first evaluated for the ability to form biofilm and produce proteolytic enzymes. For the non-vertebrate in vivo model, *G*. *mellonella* insects were infected with 10^5^, 10^6^, and 10^7^ yeasts into the last left proleg and monitored over seven days. For the vertebrate in vivo model, C57BL/6 female mice were infected with 5 × 10^6^ yeasts into the lateral tail vein and evaluated at 3 and 20 days post-infection, corresponding to the day of EAE induction and 17 days after EAE induction, respectively. For the in vitro model, microglia cells (BV-2 cell line) were infected with 5 × 10^5^ yeasts and evaluated after 6 and 24 h post-infection.

Initially, assays of biofilm formation and proteolytic activity of *Candida* strains were performed. After that, two in vivo experimental models were employed, including a non-vertebrate model with *G. mellonella* insect and a vertebrate model with *M. musculus* mice. In addition, an in vitro model, using microglia cell lineage derived from mice, was performed in this study. This experimental design is illustrated below, in Figure 1.

### 2.4. Biofilm and Proteolytic Activity

Each *Candida* strain was standardized (10^7^ cells/mL) in yeast nitrogen base broth (YNB, Difco, Detroit, MI, USA) with 100 mM glucose and 0.2% bovine serum albumin (BSA, Sigma-Aldrich, St. Louis, MO, USA). Then, 2 mL aliquots of this suspension were added to the wells, and they were incubated at 37 °C under agitation (95 rpm) to form the biofilms on the bottom of 24-well plates. After 1 h 30 min (initial adherence), 4 h, and 24 h of biofilm formation, the proteolytic activity and the number of viable cells were determined. To quantify the proteolytic activity, 100 µL of the supernatant from each well was placed in contact with 400 µL of 0.1 M sodium citrate buffer (pH 3.2) containing 1% BSA. This mixture was incubated for 1 h, and after this period, 500 µL of 5% trichloroacetic acid was added to stop the proteolytic reaction. The mixture was incubated at 4 °C for another 1 h, and 100 µL was transferred to a UV-transparent 96-well plate for reading in the spectrophotometer at 280 nm. To determine the number of *Candida* viable cells, the biofilm was detached using an ultrasonic homogenizer (Sonoplus HD 2200, Bandelin Electronic, Berlin, Germany) at 7 W for 20 s. Serial dilutions were prepared and plated on Sabouraud dextrose agar followed by incubation at 37 °C for 48 h. Then, the number of colony-forming units (CFU/mL) was calculated.

### 2.5. G. mellonella Assay

Larvae of *G. mellonella* were infected with 10 μL of yeast inoculum (10^7^, 10^8^, and 10^9^ cells/mL) into the last left proleg and incubated in the dark at 37 °C, with no food. Then, the larvae were monitored over seven days to determine the survival curve and health index. Larvae injected with PBS were used as control groups. The survival rate was calculated by the number of dead larvae recorded daily. The larvae were considered dead when they displayed no movement in response to touch. The health index was determined according to a pathological scoring system proposed by Loh et al. [36] for the following attributes: larvae activity (0: no movement, 1: movement with minimal stimulation, 2: movement with stimulation, and 3: movement without stimulation); cocoon formation (0: without cocoon, 0.5: incomplete cocoon, and 1: complete cocoon); melanization (0: complete melanization, 1: dark points in brown larva, 2: more than three points in beige larva, 3: less than three points in beige larva, and 4: no melanization); survival (0: dead larva, and 2: alive larva). All the scores together corresponded to the health index.

### 2.6. Candida Infections in Mice

C57BL/6 female mice (8–9 weeks old) were infected with 0.1 mL of yeast inoculum (5 × 10^7^ viable yeasts/mL) into the lateral tail vein three days before EAE induction, and the tissue samples were collected 3 and 20 days post-infection, that is, 17 days after encephalomyelitis induction, at the peak of clinical manifestations. The acquisition of samples was preceded by anesthesia with ketamine/xylazine and perfusion with 5 mL of phosphate-buffered saline (PBS). Mice injected with PBS were used as control groups.

### 2.7. EAE Induction and Clinical Evaluation

EAE was induced by subcutaneous immunization with 100 µg of myelin oligodendrocyte glycoprotein peptide (MOG_35–55_, Genemed Synthesis Inc., San Antonio, TX, USA) emulsified in 25 µL of Complete Freund’s Adjuvant (Sigma-Aldrich, St. Louis, MO, USA) containing 2 mg/mL of *Mycobacterium tuberculosis* (Difco, Detroit, MI, USA). Mice also received two intraperitoneal doses (200 ng each) of *Bordetella pertussis* toxin (Sigma-Aldrich), one on the day of immunization and the other 48 h later. Disease manifestation and severity were daily checked by body weight loss and clinical score. Clinical scores were defined according to the following criteria: 0—no symptoms; 1—limp tail; 2—loss of hip tone; 3—partial hind leg paralysis; 4—complete hind leg paralysis; and 5—quadriplegia/death. Prevalence represents the percentage of mice in the group that developed the disease, which was assessed every day by using the presence of a clinical score manifestation, during all experimental protocol.

### 2.8. Fungal Load Determination

Samples from the spleen, kidneys, and CNS were collected, weighed, and dissociated in 1 mL of sterile PBS using a sterile plastic pestle for microtube. The 0.1 mL aliquots from these homogenates were plated on Sabouraud dextrose agar (Becton Dickinson and Company) and then incubated for 3 days at 37 °C. The number of colony-forming units was counted, and the results were normalized per gram of tissue and logarithmized.

### 2.9. Histopathological Evaluation

Lumbar spinal cord samples were fixed in 10% neutral buffered formalin for 24 h, washed in water for 16–18 h, and immersed in 70% ethyl alcohol. The samples were dehydrated in series of absolute ethyl alcohol and xylol and embedded in Paraplast Plus (Sigma-Aldrich). The 4 µm thick histological sections were stained with HE (hematoxylin–eosin), and inflammatory infiltrate was evaluated in a Nikon microscope (Nikon Corporation, Melville, NY, USA).

### 2.10. RT-PCR

Inguinal lymph nodes frozen in liquid nitrogen were used for RNA extraction with Trizol reagent (Invitrogen, Carlsbad, CA, USA), and cDNA synthesis (High-Capacity RNA-to-cDNA converter kit, Applied Biosystems, Foster City, CA, USA) was performed according to the manufacturer’s recommendations. The expression of *Tbx21* (Mm00450960_m1), *Rorc* (Mm01261022_m1), *Gata3* (Mm00484683_m1), and *GAPDH* (Mm99999915_m1) genes were analyzed by real-time PCR using the Taqman system (Applied Biosystems) according to the manufacturer’s recommendations. Gene expression was based on the levels of *GAPDH* reference gene and represented as relative fold change (2^−ΔΔCt^) using the control group as a calibrator.

### 2.11. Splenic Cell Cultures

Spleens were gently dissociated in PBS and resuspended in RPMI 1640 medium (Sigma-Aldrich) supplemented with 10% fetal bovine serum (FBS, Gibco, Invitrogen, Waltham, MA, USA), 1% L-glutamine (Sigma-Aldrich), and 2% antibiotic/antimycotic (Sigma-Aldrich). Cultures (5 × 10^6^ cells/mL) from non-EAE mice were stimulated with concanavalin A (20 µg/mL, Sigma-Aldrich) and from EAE mice were stimulated with yeasts killed by heat and pressure at a 5:1 ratio (five yeasts for each cell) or MOG_35–55_ (20 µg/mL, Genemed Synthesis Inc., San Antonio, TX, USA). After 48 h in a humidified incubator at 37 °C and 5% CO_2_, the cell-free supernatants were stored at −80 °C for subsequent cytokine quantification.

### 2.12. Isolation of CNS Mononuclear Cells

Brain and spinal cord were dissociated in PBS and digested with 2.5 mg/mL collagenase D (Roche Applied Science, Indianapolis, IN, USA) and 100 μg/mL DNAse (Sigma-Aldrich) in 4 mL RPMI for 45 min at 37 °C. The cell suspensions were washed in Hank’s Balanced Salt Solution (HBSS) by centrifugation at 450× *g* at 4 °C for 7 min and resuspended in 30% gradient of Percoll (GE Healthcare, Uppsala, SWE), gently placed over 70% gradient in 15 mL tubes and then centrifuged at 950× *g* at 4 °C for 20 min with centrifuge brakes deactivated. After centrifugation, the ring containing mononuclear cells was collected, washed with RPMI, and resuspended in staining buffer (PBS with 5% FBS and 0.1% NaN_3_) for flow cytometric analysis.

### 2.13. Microglial Cell Culture

Microglia (BV-2 cell line) was employed to analyze the local inflammatory potential of these non-*albicans Candida*. The BV-2 cell line (BCRJ code 0356) was plated on 24-well plates at 5 × 10^5^ cells per well in DMEM High Glucose medium (Gibco) supplemented with 20% heat-inactivated FBS (Gibco), 1% of sodium pyruvate (Sigma-Aldrich), and 2% antibiotic/antimycotic (Sigma-Aldrich). After 2.5 h incubation for adherence, *Candida* spp. were added (5 × 10^5^ yeast per well), and the plate was incubated in a humidified incubator at 37 °C and 5% CO_2_. The supernatants were collected after 6 and 24 h of incubation for cytokine quantification, whereas the cells were collected only after 6 h for cytometric analysis.

### 2.14. Cytokine Quantification

Cytokine production by the spleen cell cultures was evaluated by cytometric bead array (CBA) in supernatants using a Mouse Th1/Th2/Th17 Cytokine Kit (BD Biosciences, San Diego, CA, USA) according to the manufacturer’s instructions. Data acquisition was performed using a FACS Canto II flow cytometer (BD Biosciences) from the Institute of Biosciences (UNESP, Botucatu, SP, Brazil), and the data were analyzed with FCAP Array 3.0 (Soft Flow Inc., St. Louis Park, MN, USA). Cytokine production by the microglia cell cultures was evaluated by enzyme-linked immunosorbent assay (ELISA) according to the manufacturers’ set kit (R&D Systems, Minneapolis, MI, USA).

### 2.15. Flow Cytometry

CNS cells were adjusted to 5 × 10^5^/tube and labeled for 30 min at 4 °C with the following anti-mouse antibodies: APC-conjugated anti-CD45 (clone 30-F11); APC-eFluor 780-conjugated anti-CD11b (clone M1/70); PerCP-Cy5.5-conjugated anti-CD3 (clone 145-2C11); PE-Cy7-conjugated anti-Ly6C (clone HK1.4); and PE-conjugated anti-Ly6G (clone 1A8) for CNS leukocyte populations and with APC-eFluor 780-conjugated anti-CD11b (clone M1/70); BB700-conjugated anti-MHCII (clone IA/IE); PE-conjugated anti-CD86 (clone GL1); and FITC-conjugated anti-CD40 (clone 1C10) for microglia activation. Data acquisition was performed using a FACS Melody (BD Biosciences, San Jose, CA, USA) (University of São Paulo, Ribeirão Preto, SP, Brazil), and the data were analyzed with FlowJo software (Becton Dickinson and Company, Franklin Lakes, NJ, USA).

### 2.16. Statistical Analysis

Normality was tested using the Shapiro–Wilk test for all data. To compare means of two groups, a t-test was used in the case of parametric data or Mann–Whitney test in the case of non-parametric data. Considering a two-way layout, interaction means were compared using an ANOVA followed by the Tukey test or Kruskal–Wallis test. Survival curves were evaluated using the Kaplan–Meier product limit estimate, and a log-rank test was used to compare the curves according to the groups. Data were analyzed using GraphPad Prism 8 (GraphPad Software Inc., San Diego, CA, USA), and values of *p* < 0.05 were considered statistically significant. Results were expressed as mean ± standard deviation (SD) to parametric data and as box ± min-to-max to non-parametric data.

## 3. Results

### 3.1. Biofilm Formation, Proteolytic Activity, and Infection of G. mellonella Showed Variable Virulence Profiles of Candida Strains

**Figure 2:**
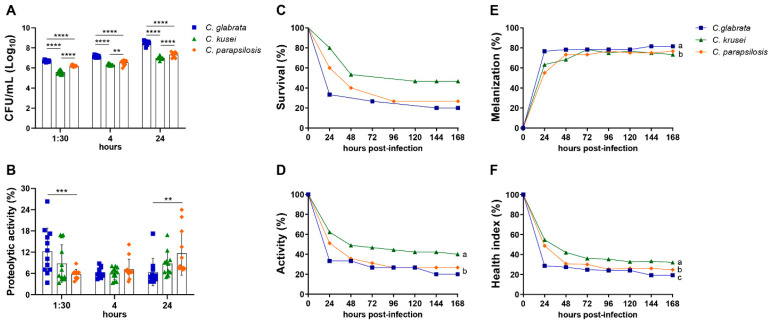
Virulence evaluation of non-*albicans Candida* species by using biofilm formation assay and *Galleria mellonella* model. Biofilm formation represented by colony-forming units (CFU/mL) (**A**) and the proteolytic activity (**B**) were analyzed at 1:30, 4, and 24 h after in vitro-inoculation of 1 × 10^7^ cells/mL of *Candida glabrata*, *C. krusei*, or *C. parapsilosis*. Larvae of *Galleria mellonella* were infected with 1 × 10^9^ cells/mL of each strain and evaluated every day for 7 days to determine the survival curve (**C**). The parameters of larval activity (**D**) and melanization (**E**) were also calculated. These data were used to determine the healthy index (**F**). The results are expressed as mean ± SD from three independent experiments (n = 12/group) and ** *p* < 0.01, *** *p* < 0.001, and **** *p* < 0.0001 (**A**,**B**). The survival curves were evaluated using the Kaplan–Meier, an product limit estimate, in two independent experiments (n = 15/group) (**C**). The results are expressed as mean from two independent experiments (n = 15/group), and different letters indicate *p* < 0.05 (**D**–**F**).

The capacity of biofilm formation increased over time for all species, but *C. glabrata* showed a significantly higher CFU in comparison to the other species at all time points (Figure 2A). The proteolytic activity of *Candida* strains was time-dependent, and *C. glabrata* showed an enhanced and faster proteolytic capacity compared to the other strains (1:30 h) (Figure 2B).

The inoculum concentration affected the survival rate of *G. mellonella* infected with each *Candida* strain. In the three analyzed species, the highest inoculum of infection caused the highest mortality rate (Figure S1A–C). The highest inoculum was then used to compare the survival curves and the healthy indexes among the three species. In the comparative analysis of survival curves, the survival rate 24 h after infection was 33% for *C. glabrata*, 60% for *C. parapsilosis*, and 80% for *C. krusei*. Although *C. glabrata* had a greater mortality rate, no statistically significant differences were detected in the survival curves compared to the other *Candida* species (Figure 2C). In relation to the health index, *G. mellonella* infected with *C. glabrata* and *C. parapsilosis* presented a significantly lower activity in comparison to the larvae infected with *C. krusei* (Figure 2D). The cocoon formation was not altered by the different species (data not shown). Concerning the melanization response, *C. glabrata* infection showed a significantly higher melanization in comparison to larvae infected with *C. parapsilosis* and *C. krusei* (Figure 2E). Therefore, the comparison of the aforementioned parameters indicated a lower and a higher health index for *C. glabrata* and *C. krusei* infected larvae, respectively (Figure 2F). The joint analysis of biofilm formation, proteolytic activity, survival curve, and healthy index indicated that *C. glabrata* was the most virulent strain.

### 3.2. Previous Infection with C. glabrata and C. krusei Strains Aggravated EAE Clinical Signs

**Figure 3:**
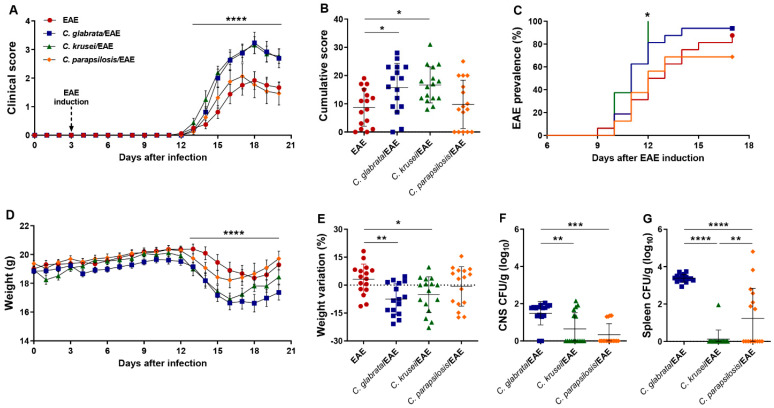
Effect of non-*albicans Candida* infection on EAE development. Female C57BL/6 mice were infected with 5 × 10^6^ viable yeasts of *C. glabrata*, *C. krusei*, or *C. parapsilosis* three days before EAE induction and evaluated for the daily clinical score (**A**), cumulative score (**B**), the percentage of disease prevalence (**C**), daily body weight (**D**), and weight variation percentage considering the initial (day 0) and the final weight (day 20) (**E**). Fungal load in CNS (**F**) and spleen (**G**) samples were evaluated on the 20th day after infection. The results are expressed as mean ± SD from four independent experiments (n = 16/group), and * *p* < 0.05, ** *p* < 0.01, *** *p* < 0.001, and **** *p* < 0.0001.

To test a possible worsening of encephalomyelitis by non-*albicans Candida* infection, we used a less severe disease model induced by lower amounts of neuroantigen (MOG) and adjuvant [22], as we had already employed to test *C. albicans*’ effect on EAE development [32]. Then, female C57BL/6 mice were intravenously infected with *C. glabrata*, *C. krusei*, and *C. parapsilosis* and then submitted to EAE induction. Despite a few differences, *C. glabrata* and *C. krusei* triggered a similar deleterious effect characterized by increased clinical score (Figure 3A), higher cumulative score (Figure 3B), and marked loss of body weight (Figure 3D,E) in comparison to the non-infected EAE control group. Disease prevalence increased steadily, reaching 87.5% (14/16) at day 17 after EAE induction in non-infected mice. Although *C. glabrata* and *C. krusei* worsen the disease, only *C. krusei* changed encephalomyelitis prevalence, triggering an early EAE onset. The percentage of sick animals in the *C. krusei*/EAE reached 100% (16/16) at day 12 and was significantly different from the non-infected EAE group (Figure 3C). The prevalence in *C. glabrata*/EAE and *C. parapsilosis*/EAE groups, 93.75% (15/16) and 68.75% (11/16), respectively, were comparable to those observed in the EAE group.

The three species were recovered from the CNS and the spleen of EAE-infected mice. Both CNS and spleen fungal burden were significantly higher in the *C. glabrata*/EAE group (Figure 3F,G).

### 3.3. Th1/Th17 Profiles Induced by C. glabrata and C. krusei Strains Persisted during EAE Development

**Figure 4:**
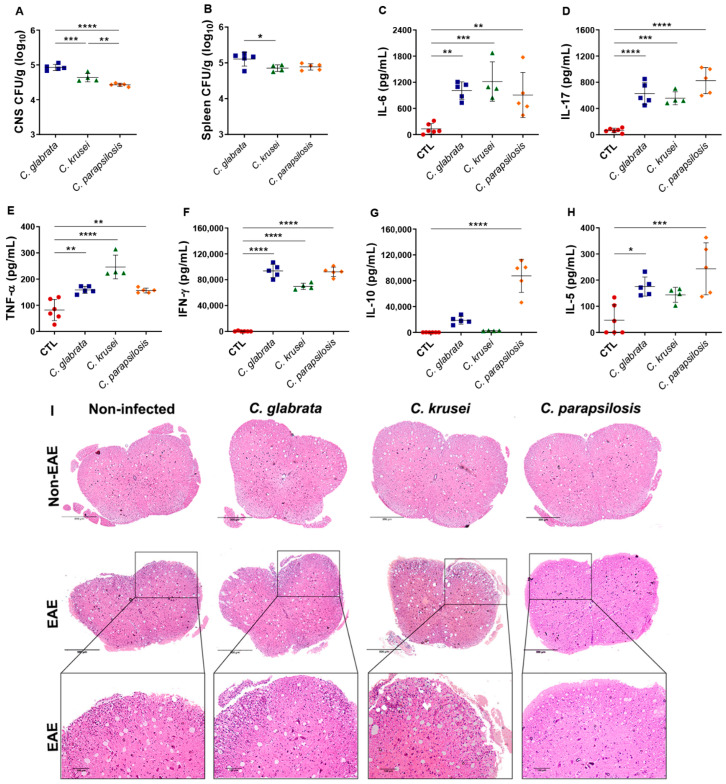
Effect of non-*albicans Candida* infection in control and EAE mice. Female C57BL/6 mice were infected with 5 × 10^6^ viable yeasts of *C. glabrata*, *C. krusei*, or *C. parapsilosis*, and fungal loads in CNS (**A**) and spleen (**B**) samples were evaluated three days later. IL-6 (**C**), IL-17 (**D**), TNF-α (**E**), IFN-γ (**F**), IL-10 (**G**), and IL-5 (**H**) levels were quantified in splenic cell cultures (5 × 10^6^ cells/mL) stimulated with concanavalin A (20 µg/mL). Representative images of the inflammatory infiltrate (**I**) observed in the lumbar spinal cord samples. The results are expressed as mean ± SD from one experiment (n = 4–6/group) and * *p* < 0.05, ** *p* < 0.01, *** *p* < 0.001, and **** *p* < 0.0001.

Healthy (control) mice were infected with *C. glabrata*, *C. krusei*, and *C. parapsilosis* and evaluated three days later. At up to 3 days of infection, no significant body weight loss was observed in any of the experimental groups (data not shown). Analogously to data observed in EAE-infected mice, the three species reached the CNS and the spleen, with *C. glabrata* being the one that showed the highest fungal burden (Figure 4A,B).

Splenic cells derived from mice infected with any of the three species and in vitro stimulated with concanavalin A produced high levels of IL-6, IL-17, TNF-α, and IFN-γ (Figure 4C–F). However, only *C. parapsilosis* was able to induce much higher levels of IL-5 and IL-10 (Figure 4G,H). Although all species reached the CNS by the third day of infection, no cellular infiltration was observed in their lumbar spinal cord samples at this time point. All EAE-mice groups, except the one infected with *C. parapsilosis*, showed an evident cellular infiltration in the white matter of the spinal cord (Figure 4I).

**Figure 5:**
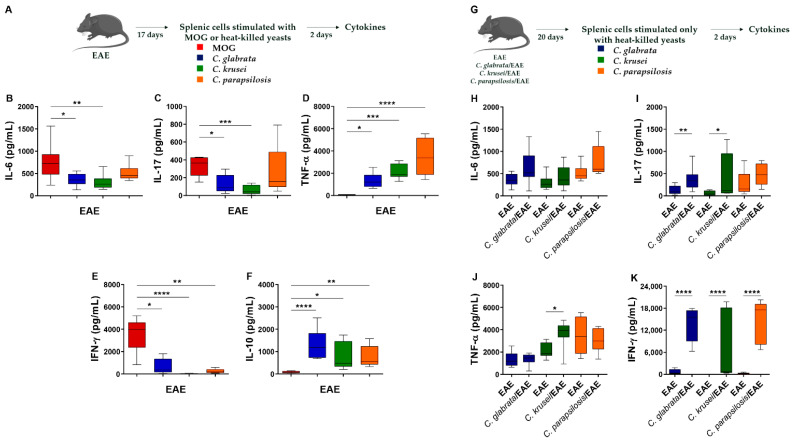
Effect of non-*albicans Candida* infection in the systemic immune response of EAE mice. Splenic cells (5 × 10^6^ cells/mL) derived from non-infected EAE mice were stimulated with MOG (20 µg/mL) or with non-*albicans Candida* heat-killed yeasts (1 cell: 5 yeasts) for 48 h (**A**). IL-6 (**B**), IL-17 (**C**), TNF-α (**D**), IFN-γ (**E**), and IL-10 (**F**) levels were quantified in cell-free supernatants. Splenic cells (5 × 10^6^ cell/mL) derived from non-infected and infected EAE mice were stimulated with non-*albicans Candida* heat-killed yeasts (1 cell: 5 yeasts) for 48 h (**G**). IL-6 (**H**), IL-17 (**I**), TNF-α (**J**), and IFN-γ (**K**) levels were quantified in cell-free supernatants. The results are expressed as box ± min to max from three independent experiments (n = 8–13/group) and * *p* < 0.05, ** *p* < 0.01, *** *p* < 0.001, and **** *p* < 0.0001.

Considering that only splenic cells derived from mice infected with *C. parapsilosis* produced IL-10 and IL-5, in addition to pro-inflammatory cytokines, three days post-infection, we analyzed if this distinct scenario persisted during EAE progression. Gene expression evaluation in draining lymph nodes did not indicate differences in *Tbx21*, *Rorc*, and *Gata3* mRNA expression among the groups (Figure S2). However, the stimulation of splenic cells with neuroantigen or Candida-derived antigens indicated clear differences. As could be expected, splenic cells from non-infected and MOG-immunized mice (Figure 5A) usually released more pro-inflammatory cytokines in response to the cognate antigen, such as IL-6, IL-17, and IFN-γ (Figure 5B,C,E), than in response to *Candida* antigen stimulation. However, higher TNF-α and IL-10 levels (Figure 5D,F) were detected in the cultures stimulated with the fungi-derived antigens.

Then, we evaluated cytokine levels produced by splenic cells from non-infected and infected EAE mice upon specific stimulation with *Candida*-derived antigens (Figure 5G). No differences were observed in IL-6 and TNF-α levels (Figure 5H,J); however, splenic cells from EAE mice infected with *C. glabrata* and *C. krusei* produced significantly higher levels of IL-17 and IFN-γ (Figure 5I,K). Only IFN-γ levels were significantly increased upon stimulation with *C. parapsilosis*-derived antigen (Figure 5K). No difference was observed in IL-10 levels (data not shown).

### 3.4. Previous Candida spp. Infection Increased Leukocyte Infiltration in the CNS of EAE Mice

**Figure 6:**
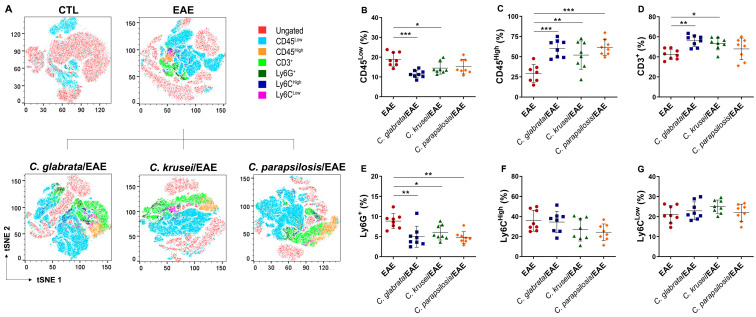
Leukocyte infiltration in the CNS of infected EAE mice. Mononuclear cells eluted from the CNS of mice with EAE were stained with anti-mouse fluorochrome conjugated monoclonal antibodies and analyzed by flow cytometry through t-distributed stochastic neighbor embedding (tSNE) plots (**A**). Percentage of CD45^Low^ (**B**), CD45^High^ (**C**), CD3^+^ gated on CD45^High^Ly6G^-^ (**D**), Ly6G^+^ gated on CD45^High^ (**E**), Ly6C^High^ gated on CD45^High^Ly6G^−^CD11b^+^ (**F**), and Ly6C^Low^ gated on CD45^High^Ly6G^−^CD11b^+^ (**G**) cells. The results are expressed as mean ± SD from two independent experiments (n = 8/group) and * *p* < 0.05, ** *p* < 0.01, and *** *p* < 0.001.

The inflammatory content of the CNS infiltrate, previously observed by histopathological analysis in EAE control mice (Figure 2I), was then characterized by flow cytometry (Figure 6A). The proportion of resident immune cells (CD45^Low^), which correspond to the resting microglia, was significantly lower in EAE mice previously infected with *C. glabrata* and *C. krusei*; this reduction was more discrete and not statistically significant in *C. parapsilosis*-infected animals (Figure 6B). Otherwise, the proportion of total leukocytes (CD45^High^) was significantly higher in EAE mice infected with any of the three species (Figure 6C). Concerning infiltrating leukocytes, an increased proportion of T lymphocytes (CD3^+^) was observed in EAE mice previously infected with *C. glabrata* and *C. krusei*, but not in animals infected with *C. parapsilosis* (Figure 6D). On the contrary, a reduction in the proportion of neutrophils (Ly6G^+^) was detected in all infected EAE mice (Figure 6E). No alterations were observed in the proportions of monocyte subsets, neither in inflammatory monocytes (Ly6C^High^) nor in patrolling monocytes (Ly6C^Low^) (Figure 6F,G).

### 3.5. C. krusei Strain Was Highly Pro-Inflammatory for Microglia

**Figure 7:**
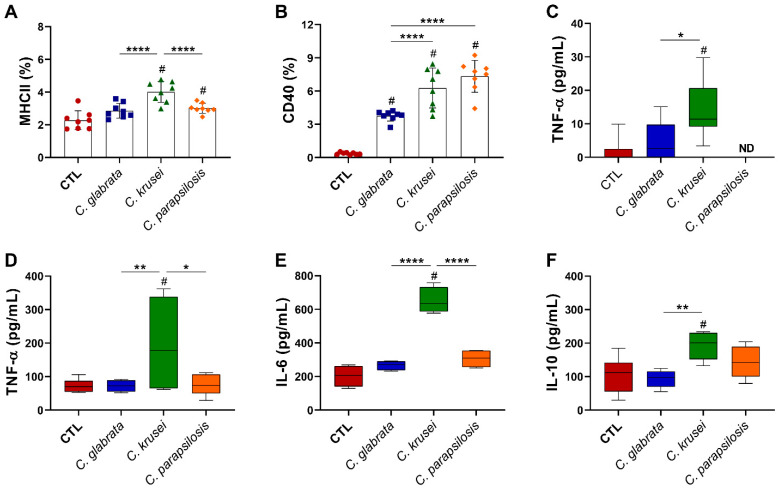
Effect of non-*albicans Candida* on microglia. Microglia cell line (BV-2, 5 × 10^5^/mL) was infected with non-*albicans Candida* viable yeasts (1 cell: 1 yeast) and evaluated after 6 (**A**–**C**) and 24 (**D**–**F**) hours. Percentage of MHCII (**A**) and CD40 (**B**) were evaluated on CD11b^+^ live (FVS^−^) and single cells (FSC-H vs. FSC-A). TNF-α (**C**,**D**), IL-6 (**E**), and IL-10 (**F**) levels were quantified in cell-free supernatants. The results are expressed as mean ± SD or box ± min to max from two independent experiments (n = 8/group), and * *p* < 0.05, ** *p* < 0.01, and **** *p* < 0.0001. # *p* < 0.05 in comparison to CTL. ND: note detected.

At 6 h after in vitro infection, microglia cells infected with *C. krusei* and *C. parapsilosis* presented a significantly higher expression of MHC II (Figure 7A), and all species displayed increased expression of CD40 co-stimulatory molecule (Figure 7B) in comparison to non-infected microglia cells (control—CTL). Concerning cytokine production at the early period, only TNF-α was detected in the culture supernatants, with the highest levels being found in *C. krusei*-infected microglia cell cultures (Figure 7C). The highest level of TNF-α (Figure 7D), in addition to IL-6 and IL-10 levels (Figure 7E,F), was observed in *C. krusei*-infected microglia cells after 24 h of culture.

## 4. Discussion

The possible association between systemic candidiasis with MS is reinforced by the fact that infectious agents, such as *Candida* spp., can cause demyelinated lesions [37]. The pathogenesis of *Candida* infection involves several virulence factors, including the capacity of biofilm formation and proteolytic enzyme production [38,39]. In comparison to *C. albicans*, it has been described that non-*albicans Candida* species are less pathogenic. This is partially associated to the reduced ability to form biofilms and lower secretion of degradative enzymes [38,40]. Despite this perception, it has been described that an increased prevalence in infections has been triggered by these species [12,13], as well as a reduced effectiveness of the most commonly used antifungal drugs against them [41]. In this sense, our study investigated the role of non-*albicans Candida* infection on encephalomyelitis development.

For this, three non-*albicans Candida* strains were selected: strains of *C. glabrata*, *C. krusei*, and *C. parapsilosis*. It is important to mention that genetic differences occur among *Candida* species and among different isolates of the same species [42]. To confirm that the selected *Candida* strains for this study were virulent, we firstly characterized these strains in relation to their capacity of forming biofilms, producing proteolytic enzymes, and infecting *G. mellonella*. All the strains were able to form biofilms and produce proteolytic enzymes; however, these characteristics were more pronounced in *C. glabrata* compared to *C. krusei* and *C. parapsilosis*. Nouraei et al. [43] analyzed the virulence factors of various non-*albicans Candida* strains isolated from patients with candidemia, verifying that 98% of the strains were capable of producing biofilms. These authors also observed proteinase activity in 100% of the non-*albicans Candida* strains, although they presented different levels of activity.

The ability to form biofilm is recognized as a specific feature of *Candida* pathogenicity, associated with the protection of fungi from external factors such as antifungal drugs and host immune defenses [44]. Regarding the latter, *Candida* biofilms alter mononuclear cell cytokine profiles, directly impacting the immune responses [45]. Proteolytic activity of *Candida* spp. is mainly mediated by a family of 10 secreted aspartyl proteinases (Sap proteins) [46]. These proteases degrade host defense peptides and E-cadherin inter-epithelial cell junctional protein, conferring resistance to the immune response and enabling the fungi to penetrate between epithelial cells [47,48]. These enzymes are also able to degrade antibodies, complement components, and cytokines [49]. According to Saroukolaei et al. [37], the secretion of fungal proteases damages the surrounding tissues and leads to inflammation; therefore, the continuous synthesis of proteolytic enzymes by fungal cells may contribute to the alterations in the CNS.

We also employed a non-vertebrate host model to characterize the virulence of *Candida* strains. The virulence of *Candida* has been traditionally investigated by using murine experimental infections. An alternative modelling that is cheaper, faster, and ethically more acceptable is the use of *G. mellonella* [50,51]. In this model, *C. glabrata* presented a higher virulence, reinforcing the aforementioned finding that the *C. glabrata* strain was more virulent than the two other non-*albicans Candida* strains used in our study. Indeed, some studies have reported a correlation between mortality rate in *Galleria* with biofilm formation [52] and proteinase activity [49,53] of the *Candida* strains. On the basis of our in vitro and in vivo assays, we proved that the strains of *C. glabrata*, *C. krusei*, and *C. parapsilosis* employed in this study showed attributes of virulence and were pathogenic for *G. mellonella*, being an important screening model for further studies in mice.

By using the EAE model, we previously demonstrated that *C. albicans* systemic infection aggravates neuroinflammation [32]. To evaluate if non-*albicans Candida* strains presented a similar effect on EAE development, C57BL/6 mice were systemically infected with strains of *C. glabrata*, *C. krusei*, and *C. parapsilosis*, and three days later, they were submitted to EAE induction. This protocol was designed to create an artificial system in which the induction of autoimmune cells specific for myelin antigens would be generated in the inflammatory environment induced by *Candida* infection. Mice infected with *C. glabrata* and *C. krusei* developed a more severe form of the disease characterized by significantly higher cumulative scores and marked body weight loss; *C. krusei* infection also increased the prevalence of the disease. Contrastingly, the infection with *C. parapsilosis* strain did not affect EAE development. These results indicated that non-*albicans Candida* infections contributed to the severity and development of encephalomyelitis; however, these effects seem to depend on the strain used, as we observed in this study.

To understand the differential effect of non-*albicans Candida* infection over EAE development, we characterized the immunological scenario established by these *Candida* strains during the moment of EAE induction, that is, three days after infection. This was considered relevant because cytokines are pivotal mediators during the neuroinflammatory process in both EAE [30] and MS [54], and *Candida* infection could interfere in the production of cytokines. On the third day after infection, the three *Candida* species were recovered from the spleen and CNS of non-EAE mice. As expected, the presence of these fungi in the spleen was concomitant with a significant production of cytokines, especially of the pro-inflammatory ones such as IL-6, IL-17, TNF-α, and IFN-γ. It is well established that these peripheral cytokines can contribute to the blood brain-barrier (BBB) breakdown process [55], which allows the migration of inflammatory cells into the CNS [56], as well as with the activation of immune cells inside the CNS itself since these mediators are able to cross the BBB [57]. Interestingly, only *C. parapsilosis*, which did not aggravate EAE, was able to induce higher levels of IL-5 and IL-10 that are mediators that have been associated with downregulation of EAE severity [58]. These results highly suggest that the cytokine profile produced in the periphery is one of the possible mechanisms by which *Candida* strains may affect the development of EAE.

The anti-*Candida* immune response is initiated upon the recognition of the pathogen-associated molecular patterns by innate immune cells that produce IL-1β, TNF-α, and IL-6, leading to effector responses mediated mainly by neutrophils and favoring the differentiation of Th1 and Th17 lymphocyte subsets [59]. These subsets produce IFN-γ and IL-17, which act on macrophages, neutrophils, and epithelial cells to further amplify the anti-*Candida* immune responses. Th1 and Th17 lymphocyte subsets, important to anti-*Candida* immune response, are also directly involved in both EAE and MS immunopathogenesis [60]. In this scenario, we could assume that together, these two responses could result in a greater availability of peripheral cytokines, their diffusion to the CNS, and the local activation of inflammatory cells. Then, we evaluated the peripheral cytokine production in response to fungal antigens during the acute phase of EAE and observed that spleen cells, derived from infected EAE mice, produced IFN-γ and IL-17 in response to the three available strains. These results indicated that there are cells specific for these strains in the periphery, even after 20 days of infection, and in response to fungi stimuli, these cells can act as cytokine-producing sources in the periphery.

Despite having reached the CNS, these *Candida* species did not induce cellular infiltration in lumbar spinal cord of non-EAE mice. Regarding the brain, we previously demonstrated that non-EAE mice infected with these non-*albicans Candida* strains presented a significant reduction in both fungal load and inflammation score when days 3 and 14 after infection were compared [34], being highly suggestive that immunocompetent mice are able to control the infection by these *Candida* strains. However, EAE mice infected with *C. glabrata* and *C. krusei* presented a higher cellular infiltration in lumbar spinal cord, and the flow cytometric analysis revealed that these two strains increased the percentage of T lymphocytes (CD3^+^) in EAE mice. These results reinforce the possibility that the anti-fungal immune response, together with the anti-MOG response, considering that they are both polarized in the Th1 and Th17 direction, could result in a higher influx of cells to the CNS, constituting, therefore, a second mechanism by which non-*albicans Candida* could aggravate EAE development. The presence of a higher amount of Th17 cells is especially relevant because this subset is clearly involved in the disruption of the BBB [61]. Even though *C. parapsilosis* was also able to induce typical Th1 and Th17 cytokines, this strain did not increase the percentage of T lymphocytes (CD3^+^) in the CNS of EAE mice and presented the lowest fungal burden in the CNS in comparison to the other two strains.

The negative correlation between the proportion of CD45^Low^ and CD45^High^ cells in the CNS of infected EAE mice and the fact that once activated, microglia trigger the expression of CD45 [62], lead us to hypothesize that microglia could be directly activated by non-*albicans Candida* strains. However, according to our data, even though all three strains were able to reach the CNS, the result of their interaction with microglial cells was distinct. We found that only *C. krusei* was able to induce a significantly higher production of cytokines and a much more accentuated expression of MHCII after co-cultivation with the BV-2 microglial cell line. The possibility that this activation of microglia is related to the morphology of *Candida* cells cannot be excluded. *C. albicans* can be found in yeast, pseudohyphae, and hyphae morphologies, whereas *C. glabrata* grows almost exclusively in the yeast morphology and *C. parapsilosis* is found in yeast and pseudohyphae morphologies [38]. The relationship between morphology and pathogenicity has been described and indicates that the yeast form is less virulent than the hyphae one [38,63]. Concerning morphology, we previously demonstrated that in microglia culture, *C. glabrata* and *C. parapsilosis* remain in the yeast form, whereas *C. krusei* grows in the pseudohyphae form [34]. In this context, our in vitro data suggest that microglia activation could be a third mechanism by which *C. krusei* could deleteriously affect EAE development and is possibly related to the fact that this strain is able to change its form from yeast to hyphae during microglia infection.

Altogether, our results demonstrated that *C. glabrata* and *C. krusei* interfered deleteriously in EAE development, initially by enhancing Th1 and Th17 responses in the periphery and allowing the increase in CD3^+^ cell influx in the CNS. We also understand that *C. krusei* would have an additional or alternative disease aggravation mechanism related to its ability to activate a pro-inflammatory profile upon interaction with microglial cells. The presence of a 100% disease prevalence in this group, 12 days after immunization with MOG, reinforces the relevance of this mechanism.

Even though we believe that these findings are contributing to emphasize the possible effect of non-*albicans Candida* infection to MS development, we are fully aware that this study has some limitations. In this sense, we would like to stress the need to evaluate more strains, including laboratory strains and clinical isolates, and to test the effect of infections in mice injected with adjuvant and pertussis toxin in the absence of MOG immunization. These procedures could shed more light to the mechanisms by which these fungi are aggravating EAE.

## 5. Conclusions

The present study indicated that strains of *C. glabrata* and *C. krusei*, similarly to *C. albicans*, aggravated EAE and could, theoretically be also deleterious to MS. In this context, our results provide an alert for the clinicians to monitor the presence of these pathogens in MS patients.

## Data Availability

The data presented in this study are available on request from the corresponding author.

## Supplementary Materials

The following supporting information can be downloaded at: https://www.mdpi.com/article/10.3390/jof8040386/s1, Figure S1: Virulence evaluation of non-*albicans Candida* species by using *Galleria mellonella* model; Figure S2: T lymphocyte subpopulations in EAE mice.
