# Supplementary material for: Systemic Infection by Non-albicans Candida Species Affects the Development of a Murine Model of Multiple Sclerosis
Source: J Fungi (Basel). 2022 Apr 10;8(4):386. doi: 10.3390/jof8040386 (PMC9032036; doi:10.3390/jof8040386)
Supplement: Supplementary file 1 [file jof-08-00386-s001.zip › jof-1643068-supplementary.pdf]

Journal of Fungi

Article

Systemic infection by non-*albicans Candida* species affects the development of a murine model of multiple sclerosis

Thais Fernanda de Campos Fraga-Silva [1*], Natália Munhoz-Alves [1], Luiza Ayumi Nishiyama Mimura [1], Larissa Ragozo Cardoso de Oliveira [2], Lívia Mara Alves Figueiredo-Godoi [3], Maíra Terra Garcia [3], Evelyn Silva Oliveira [1], Larissa Lumi Watanabe Ishikawa [1], Sofia Fernanda Gonçalves Zorzella-Pezavento [1], Vânia Luiza Deperon Bonato [4], Juliana Campos Junqueira [3], Eduardo Bagagli [1] and Alexandrina Sartori [1,2]

[1] Department of Chemistry and Biological Sciences, Institute of Biosciences, São Paulo State University (UNESP), Botucatu 18618-689, Brazil; natalia.mnhz@gmail.com (N.M.-A.); luizamimura@gmail.com (L.A.N.M.); soliveira.evelyn@gmail.com (E.S.O.); larissalumi@gmail.com (L.L.W.I.); szorzella@yahoo.com.br (S.F.G.Z.-P.); eduardo.bagagli@unesp.br (E.B.); alexandrina.sartori@unesp.br (A.S.)

[2] Postgraduate Program in Tropical Disease, Botucatu Medical School, São Paulo State University (UNESP), Botucatu 18618-687, Brazil; larissa.ragozo@unesp.br

[3] Institute of Science and Technology, São Paulo State University (UNESP), Sao Jose dos Campos 12245-000, Brazil; livia_mafigueiredo@yahoo.com.br (L.M.A.F.-G.); maira.garcia@unesp.br (M.T.G.); juliana.junqueira@unesp.br (J.C.J.)

[4] Department of Biochemistry and Immunology, Ribeirao Preto Medical School, University of Sao Paulo (USP), Ribeirao Preto 14049-900,Brazil; vlbonato@fmrp.usp.br (V.L.D.B.)

* Correspondence: thaisfragasilva@gmail.com

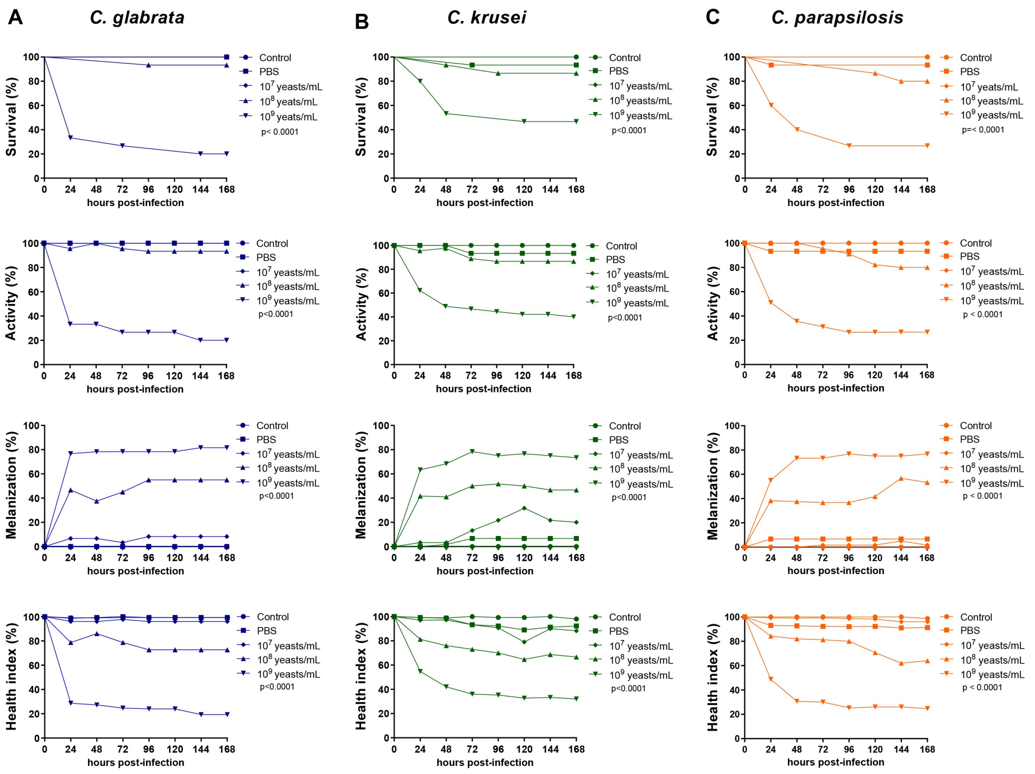

Supplementary Figure S1. Virulence evaluation of non-*albicans Candida* species by using *Galleria mellonella* model. Larvae of *Galleria mellonella* were infected with 3 different inoculum of *Candida glabrata* (**A**), *C. krusei* (**B**) and *C. parapsilosis* (**C**) and evaluated every day for 7 days to determine the survival curve. The parameters of larval activity and melanization were also calculated. These data were used to determine the healthy index. The survival curves were evaluated using Kaplan-Meier in two independent experiments (n= 15/group). The results are expressed as mean from two independent experiments.

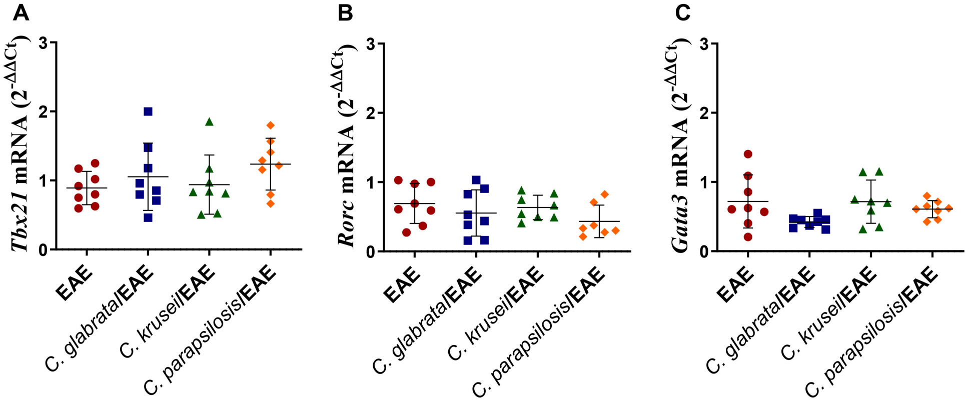

Supplementary Figure S2. T lymphocyte subpopulations in EAE mice. Female C57BL/6 mice were infected with 5×10^6 viable yeasts of *C. glabrata*, *C. krusei* or *C. parapsilosis* three days before EAE induction and evaluated for relative expression of *Tbx21* (**A**), *Rorc* (**B**) and *Foxp3* (**C**) mRNA in inguinal lymph nodes on the 17th day after EAE induction. The results are expressed as mean ± SD; n= 8/group, one representative experiment of two.
